# Boric Acid in Milk Replacer as a Health Enhancer and Growth Promoter for Lambs in the Suckling Period

**DOI:** 10.1007/s12011-024-04214-4

**Published:** 2024-05-17

**Authors:** Soner Uysal, Mehmet Akif Yoruk

**Affiliations:** 1 Department of Animal Nutrition and Nutritional Diseases, Faculty of Veterinary Medicine, Ataturk University, Erzurum, 25240 Turkey; 2 Department of Animal Nutrition and Nutritional Diseases, Faculty of Veterinary Medicine, Ondokuz Mayıs University, Samsun, 55139 Turkey

**Keywords:** Boric acid, Lamb health, Immune system, Suckling period, Gene expressions

## Abstract

This study was performed to investigate the effects of boric acid supplementation in milk replacer of lambs in the suckling period on performance, biochemical parameters, the antioxidant system, fecal culture, and expression of some genes. During the suckling period, 60 lambs (4 days old) were randomly given four levels of boric acid (0, 30, 60, and 90 mg/kg body weight) via milk replacer for 57 days. The lambs supplemented with boric acid had a higher weight gain and better feed conversion ratio. Boric acid supplementation quadratically increased serum triglyceride, total protein, alkaline phosphatase, serum antioxidant activity and oxidative stress biomarkers, and fecal flora and decreased IL1β, IL10, iNOS, NF-kB, and TNF-α gene expressions. The effect of boric acid on rumen papilla development could not be determined since the animals were not slaughtered. In conclusion, the use of boric acid to lambs in the suckling period improved the average weekly body weight gain and feed conversion efficiency, positively affected some biochemical parameters, antioxidant system, and intestinal flora, and also affected gene expressions related to the immune system. Boric acid supplementation had a beneficial effect on the health and growth of suckling lambs.

## Introduction

Animal products are highly significant for nutrition and food security, contributing a significant portion of global protein consumption [1]. The demand for animal products is increasing in parallel with increasing global population and income levels and it is projected that this will increase from 48 to 57% between 2005 and 2050 [2]. Sheep provide meat, milk, and wool and are considered as a significant source of animal protein [3]. Thus, it is crucial that the health of newborn lambs is improved. Today, neonatal lamb mortality remains an important global animal welfare problem [4]. Since the transfer of antibodies from the placenta of ruminants to the offspring is prevented, the immunity of newborns against pathogens depends entirely on the intestinal absorption of immunoglobulins consumed in colostrum from the mother [5]. The immune system response is one of the mechanisms developed to defend against environmental infectious challenges. Animals create an immune response by secreting cytokines (interleukin, interferon γ, tumor necrosis factor) against stress factors [6]. The development and health of neonatal ruminant animals are significantly influenced by intestinal microbes [7]. Intestinal bacterial imbalance, for instance, has been linked to mortality, diarrhea, dehydration, growth retardation, and disrupted nutritional digestion and absorption. In order to support the growth and health of pre-wean ruminants, research that focuses on the creation of an ideal gut microbiota would be helpful [8]. Pathogenic microorganisms that cause diarrhea in lambs, especially in the neonatal period, include *Escherichia coli* (*E.coli*) and *Clostridium perfringens* [9].

Known to be present in water, rocks, and soil, boron (B) is not naturally found in pure form [10]. Its most common form (96%) is boric acid [11]. Boron is described as playing an important role in the biological, metabolic, and physiological processes of plants and animals [12]. It is well recognized that boron is crucial to the immune and antioxidant defense systems as well as the metabolism of minerals such as calcium, phosphorus, and magnesium, which contribute to bone formation [13]. Eventually, owing to its complex influence on the development of numerous diseases, boron has attracted the interest of researchers [14]. In recent years, many studies have been conducted to investigate the antimicrobial and antifungal activities of boron and the effects of this mineral on the antioxidant and immune systems, as well as mineral and energy metabolisms in animals and humans [15–23]. Studies on the use of boric acid report that boric acid is an inexpensive boron derivative [20]. Available data on the lethality of boron is limited, so further studies are needed. The toxic effects of boron and its compounds on the body have not been adequately investigated, especially at the tissue level [11]. It is also reported that some boron derivatives (boric acid, borax, colemanite, and ulexite) may provide protection against the toxicity of heavy metals [24].

When determining the effect of additives used in animal nutrition on health and productivity, it is important to evaluate blood serum metabolites (liver enzymes, lipid and protein status, oxidant/antioxidant balance, etc.), fecal flora, and immune system [8, 11]. Although there are many studies on the addition of boric acid and other boron derivatives to animal diets, no references have been found in the literature regarding its use of lambs in the suckling period. In this study, it was aimed to determine the effect of boric acid on animal health and productivity, performance, blood biochemistry, antioxidant system, fecal flora, and gene expressions related to the immune system to lambs in the suckling period.

## Materials and Methods

### Ethical Approval

This experiment was carried out in a privately owned sheep farming enterprise in Erzurum province, located in the north-east of Turkey. All processes were ethically approved by the Atatürk University Animal Experiments Local Ethics Committee.

### Boric Acid and Preparation

Boric acid was supplied from a commercial company (TEKKIM, extra pure boric acid, TK.020.100.01002, Nilufer-Bursa, Turkey). The boric acid we used in the experiment was in solid form and we dissolved it thoroughly in the milk replacer (Ovilac Eurovo Milk Replacer, Societe Laitiere De Retiers À Retiers 35,240, Retiers, France) before giving it to the lambs. The boric acid levels used in the study are much lower than the LD50 levels (2660–4100 mg/kg).

### Animals, Diets, and Management

In total, 60 four-day-old lambs of the Lacaune breed were randomly distributed into four groups, each of 15 lambs (7 females and 8 males). The floor space per lamb was 1.24 m^2^. The pens in which the lambs were housed were well ventilated, and the floors, feeders, and drinkers were cleaned on a daily basis. Roughage and concentrate feed were provided in separate feeders. The milk replacer consumed by the lambs was given to each animal separately in individual feeding bottles. The four groups of lambs were fed individually on different milk treatments as follows: C (control)—100% milk replacer, B30—milk replacer + 30 mg/kg body weight (BW) of boric acid, B60—milk replacer + 60 mg/kg BW of boric acid, B90—milk replacer + 90 mg/kg BW of boric acid. The boric acid doses used in the study were calculated taking into account previous studies conducted on animals [25].

The lambs consumed lamb starter (Abalioğlu Karagöz Lamb Starter Feed, Mersin, Turkey) and alfalfa ad libitum. The nutritional composition of the lamb diet is as shown in Table 1. An amount of 125 g of milk replacer was dissolved in 1 L of water by stirring. Thereafter, the milk replacer was heated at 45–50 °C for 10–15 min with constant stirring. The milk replacers were given to the lambs after being cooled to 37 °C. Lambs consumed 10% of their daily live weight in total milk replacer until they were 60 days old. They were fed with milk replacer 5 times a day in the 1st week, 4 times a day in the 2nd and 3rd weeks, 3 times a day in the 4th week, and twice a day from the 5th week to the 60th day.

**Table 1:** Nutritional composition of lambs’ diet

Nutrient contents (%)	Milk replacer	Lamb starter feed	Alfalfa
Dry matter	89	89	88
Crude protein	23	17	20.2
Crude fiber	0.1	9	–
Ether extract	25.5	2.8	1.75
Crude ash	6.5	8.6	7.02
ADF	–	–	34.3
NDF	–	–	44.4

*ADF* acid detergent fiber, *NDF* neutral detergent fiber

### Performance

During the study, the daily feed intake (FI) of the lambs, the milk replacer consumed, and the remaining feed in the feed troughs were weighed and recorded every morning. Each lamb was weighed on a weekly basis, at 8:00 am using a digital balance with 100 g precision. After this weighing, the amount of boric acid they would consume in the next week was calculated and given accordingly. Using FI and BW, the average weekly weight gain (AWWG) and feed conversion ratio (FCR) were computed.

### Sampling and Measurements

Fecal samples were taken from all animals a total of 6 times at 10-day intervals per animal and were used for microbiological analyses. The feces were collected directly from the rectum, and the collected samples from the rectum were placed in individual sterile stool containers and stored in deep freezers at − 80 °C until being used for analysis [26].

One gram from each fecal sample was diluted up to 10^−6^ in 9 mL of either sterile physiological saline to be used for the enumeration of total bacteria, *C. perfringens*, and total coliform bacteria. After being mixed on a vortex mixer, viable bacteria in the dilutions were inoculated onto solid media using the spread plate method (plate count agar medium, tryptose sulfite cycloserine medium, and violet red bile medium). The media were incubated for 24 h at 37 °C under aerobic conditions for total bacteria and total coliform bacteria and for 24 h at 37 °C under anaerobic conditions for *C. perfringens*. Typical colonies which had grown in the incubated petri dishes to an approximate diameter of 0.5 mm were counted and quantified in log10 (CFU/g).

Blood samples were collected on day 60 of the study, at 9.00 am, by jugular venipuncture into blood serum tubes (BD Vacutainer SST II Advance) to be used for serum biochemical tests. The blood samples were centrifuged for 10 min at 1000 g and 4 °C to extract serum. Serum total protein (TP), albumin, cholesterol, triglyceride, high-density lipoprotein (HDL), low-density lipoprotein (LDL), aspartate aminotransferase (AST), alanine aminotransferase (ALT), alkaline phosphatase (ALP), and gamma-glutamyl transferase (GGT) levels were determined with the aid of a Randox RX model autoanalyzer (County Antrim, UK).

Malondialdehyde (MDA) and glutathione (GSH) levels were determined as described by Placer et al. [27] and Sedlak and Lindsay [28], respectively. Serum total oxidant status (TOS) levels were determined using an automated technique (TOS test kit, Rel Assay Diagnostic, Gaziantep, Turkey) developed by Erel [29]. Serum total antioxidant status (TAS) levels were also determined as described by Erel [30].

Total RNA isolation was performed on blood serum samples from all lambs of experimental and control groups with QIAzol Lysis Reagent (Qiagen, Cat: 79,306, Hilden, Germany) according to the manufacturer’s instructions. After total RNA isolation, the RNA concentration was measured by using a NanoDrop spectrophotometer (Epoch Microplate Spectrophotometer, Radnor, PA). Later on, the quality of total RNA samples was assessed regarding DNA contamination by using gel electrophoresis. cDNA synthesis was performed using QuantiTect Reverse Transcription (Qiagen, Cat: 330,411, Germany) from total RNA according to the manufacturer’s instructions.

In the measurement of selected genes (IL 1β, IL6, IL10, iNOS, NF-kB, TNF-α), the master mix was prepared using SYBR Green in real-time PCR experiments as follows: SYBR Green 2X Rox Dye Mastermix (Qiagen), forward and reverse primers, cDNAs as templates, and nuclease-free water. After the master mixes were prepared, the samples were placed in the RT–qPCR (Bio-Rad, Hercules, CA) device and the results were evaluated according to the 2^−ΔΔC^_T_ method [31] and thus the expression values of the genes were calculated. The primer sequences of the relevant genes are given in Table 2.

**Table 2:** Primer sequences of genes

Primer	Sequences 5′-3′	Accession no
IL1β	F: GTGGTGCAAGTATGAGCTGGA R: GCTTACAGAAGCGCTGGGGAA	DQ890157.1
IL6	F: TGCAGTCCTCAAACGAGTGG R: CCGCAGCTACTTCATCCGAA	NM_001009392.1
IL10	F: CCCAGTCTCTGCTGGATGAC R: CAGAAAACGATGACAGCGCC	NM_001009327.1
iNOS	F: AAGTGGTATGCTCTGCCAGC R: CCCATGTACCACCCGTTGAA	AF223942.1
NF-kB	F: GGCAGGATTGTGAACCAAAC R: CTTGAGATGGACGCAGAGGA	XM_027966801.2
TNF-α	F: CTGGTTCAGACACTCAGGTCA R: CATTGGCATACGAGTCCCCC	NM_001024860.1

*IL* interleukin, *iNOS* inducible nitric oxide synthase, *NF-κB* nuclear factor kappa, *TNF-α* tumor necrosis factor

The performance and biochemical and microbiological data were first tested for normality using the Shapiro–Wilk test. Log transformation was performed for the microbiological data. The data were subjected to one-way analysis of variance by applying the mixed model (Proc. MIXED) of the Statistical Analysis System package program (SAS, 2002). In the linear model,

$$\left(yijk=\mu +Bi+Tj+(B*T)ij+eijk, \left[N (\sigma , \mu ; 0, 1)\right]\right),$$

showing the impact of boron supplementation on the investigated variables; *y* is a parameter, *µ* is the population mean, *B* is the boron level, *T* is the time, and *e* is the experimental error, as a repeated measures. Lamb within the group was the random term. Statistical analyses included the biological response to presence and increased boric acid supplementation, control vs. boron assessment with the orthogonal contrast option, and the linear and parabolic effects with the polynomial contrast option. Differences between the group means were determined with the least significant differences (LSD) test. Differences arising from boron supplementation were considered significant at the *P* ≤ 0.05 level.

## Results

### Performance

The effects of dietary supplementation with boric acid on the growth performance of lambs in the suckling period are summarized in Table 3. The results show that there was a quadratic effect on AWWG due to very high gains in the B60 group (*P* < 0.05). In addition, it was determined that FCR of the groups supplemented with 60 and 90 mg/kg BW of boric acid was better than that of the control group (*P* < 0.005).

**Table 3:** The effects of dietary supplementation with boric acid on the growth performance of lambs in the suckling period during the experimental duration

	AB (kg)	ABW (kg)	FI (g)	AWWG (g)	FCR
C	4.61 ± 0.19	9.85 ± 0.33	3630.1 ± 150.6	1369.2 ± 61.6^b^	3.20 ± 0.18^a^
B30	4.86 ± 0.23	10.17 ± 0.35	3675.8 ± 152.3	1418.3 ± 44.9^ab^	2.91 ± 0.16^ba^
B60	4.55 ± 0.19	9.91 ± 0.36	3734.2 ± 155.2	1517.5 ± 46.8^a^	2.46 ± 0.08^c^
B90	4.70 ± 0.14	10.03 ± 0.32	3680.2 ± 151.3	1384.2 ± 53.0^b^	2.75 ± 0.14^b^
Control vs. boron	NS	NS	NS	NS	0.001
Linear	NS	NS	NS	NS	0.001
Quadratic	NS	NS	NS	0.05	0.024

± : standard error means

^a,b^Means with different superscripts in the same row indicate significant differences (*P* < 0.05)

*AB* average birth weight, *ABW* average body weight, *FI* feed intake, *AWWG* average weekly weight gain, *FCR* feed conversion ratio, *NS* not significant, *C* control, *B30* 30 mg/kg BW of boric acid, *B60* 60 mg/kg BW of boric acid, *B90* 90 mg/kg BW of boric acid

### Blood Biochemistry

Some serum biochemical parameters of lambs in the suckling period were affected by boric acid supplementation (Table 4) (*P* < 0.05). There was a quadratic effect on TP with the B60 treatment being substantially lower than other treatments (*P* < 0.05). In addition, there was a significant quadratic effect of boric acid on serum ALP in that all boric acid treatments were elevated above the control (*P* < 0.05).

**Table 4:** The effect of dietary supplementation with boric acid on serum biochemical parameters of lambs in the suckling period

Parameter	Dose (mg/kg)				Effect, *P* > *F*		
	C	B30	B60	B90	Control vs. boron	Linear	Quadratic
Lipids							
TG (mg/dL)	38.6 ± 3.9^ab^	27.4 ± 2.9^b^	31.4 ± 2.5^ab^	39.4 ± 6.0^a^	0.2204	0.7216	0.0222
CHOL (mg/dL)	43 ± 3.1	36.7 ± 3.6	35.7 ± 2.7	36.9 ± 4	0.1026	0.2133	0.2784
HDL (mg/dL)	26.6 ± 1.5	23.2 ± 2.2	23.7 ± 1.8	23.1 ± 2.7	0.1816	0.2897	0.5121
LDL (mg/dL)	8.99 ± 1.63	8.04 ± 1.92	5.69 ± 1.54	6.23 ± 1.14	0.2061	0.1388	0.6381
Proteins							
TP (g/dL)	6.05 ± 0.16^a^	5.83 ± 0.13^ab^	5.51 ± 0.13^b^	6.00 ± 0.18^a^	0.1213	0.4786	0.0221
ALB (g/dL)	2.89 ± 0.07	2.98 ± 0.06	2.87 ± 0.07	2.94 ± 0.08	0.6687	0.9339	0.9261
Enzymes							
GGT (U/L)	109 ± 5	110 ± 7	117 ± 10	96 ± 6	0.8429	0.3001	0.1344
ALT (U/L)	12.4 ± 1.4	16.7 ± 1.3	14.3 ± 2	16.2 ± 1.8	0.0879	0.2277	0.4733
AST (U/L)	102 ± 8	117 ± 7	162 ± 27	155 ± 36	0.1220	0.0586	0.6329
ALP (U/L)	327 ± 43^c^	545 ± 59^a^	499 ± 62^ab^	379 ± 52^bc^	0.0225	0.6533	0.0029

± : standard error means

^a–c^Means with different superscripts in the same row indicate significant differences (*P* < 0.05)

*TG* triglyceride, *CHOL* cholesterol, *HDL* high-density lipoprotein, *LDL* low-density lipoprotein, *TP* total protein, *ALB* albumin, *GGT* gamma-glutamyl transferase, *ALT* alanine aminotransferase, *AST* aspartate aminotransferase, *ALP* alkaline phosphatase

### Antioxidant System

**Fig. 1:**
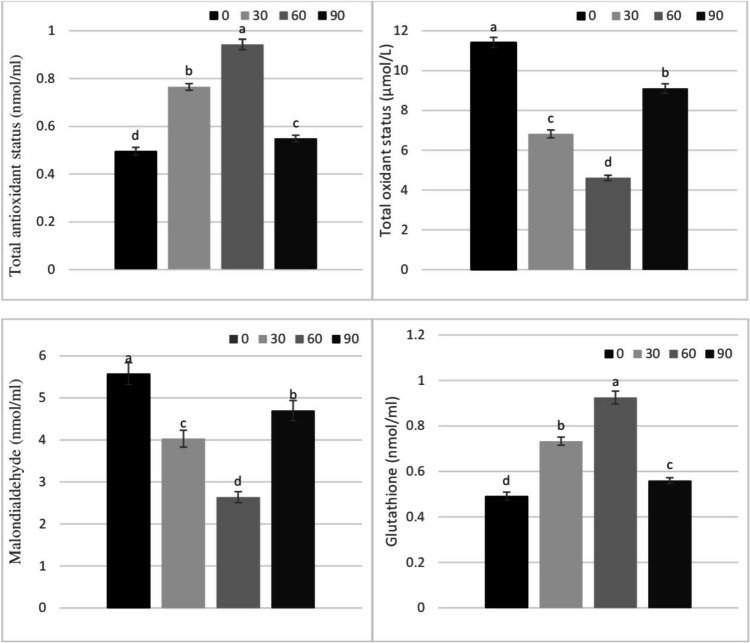
The effect of dietary supplementation with boric acid (mg/kg BW) on serum antioxidant activity and oxidative stress biomarkers in suckling lambs. Letters above error bars (a–d) indicate statistical significance

The effects of boric acid on the serum antioxidant activity and oxidative stress biomarkers of lambs in the suckling period are summarized in Fig. 1. As can be seen from the figure, there are statistical differences between the TAS of the groups (*P* < 0.05). The serum TAS levels, from the greatest to the lowest, were determined in groups B60, B30, B90, and C, respectively. According to the results obtained, it was determined that the TOS was lower in the groups where boric acid was used compared to group C (*P* < 0.05). Significant differences were observed between the control group and the groups that received dietary boric acid for serum MDA levels. Lower MDA levels were observed particularly in group B60 (*P* < 0.05). In addition, the results showed that serum GSH levels were higher in group B60 than in group C (*P* < 0.05).

### Fecal Flora

The effects of boric acid supplementation on the total fecal bacterial counts of lambs in the suckling period are summarized in Table 5. As can be seen in the table, dietary supplementation with boric acid affected in terms of bacteria examined and total bacterial load. Total coliform bacteria counts decreased with increased levels of boric acid in the diet (*P* < 0.05). Similarly, clostridial counts decreased with increased boric acid levels (*P* < 0.05). In addition, the results showed that the number of total bacteria in the feces was higher in the groups that received dietary boric acid supplementation, compared to group C.

**Table 5:** The effect of dietary supplementation with boric acid on total fecal bacterial counts of lambs in the suckling period

Bacteria	Dose (mg/kg)				Effect, *P* > *F*		
	C	B30	B60	B90	C vs. B	Linear B	Quadratic B
Total coliform, log CFU/mL^a,b,c^	7.76 ± 0.06^a^	7.56 ± 0.05^b^	7.61 ± 0.05^b^	7.40 ± 0.04^c^	0.0001	0.0001	0.9510
Clostridia, log CFU/mL^b,d,e^	7.33 ± 0.05^a^	7.24 ± 0.05^ab^	7.21 ± 0.03^b^	7.20 ± 0.03^b^	0.0068	0.0088	0.2976
Total bacteria, log CFU/mL^a,b,c^	7.73 ± 0.08^b^	8.11 ± 0.07^a^	7.98 ± 0.06^a^	8.10 ± 0.06^a^	0.0001	0.0003	0.0390

± : standard error means

^a–c^Means with different superscripts in the same row indicate significant differences (*P* < 0.05)

### Gene Expressions

The effects of boric acid supplementation on the immune system-related gene expressions of lambs in the suckling period are summarized in Table 6. The results of the study showed that IL1β, IL6, IL10, iNOS, NF-kB, and TNF-α gene expressions were generally lower in the boric acid-treated groups compared to group C (*P* < 0.05).

**Table 6:** Expression of IL1, IL6, IL10, iNOS, NF-kB, and TNF-α in the blood serum of the control and boric acid-treated lambs in the suckling period

Parameter	Dose (mg/kg)				Effect, *P* > *F*		
	C	B30	B60	B90	C vs. B	Linear B	Quadratic B
IL1β	1.6 ± 0.035^a^	0.783 ± 0.041^b^	0.633 ± 0.015^bc^	0.547 ± 0.014^c^	0.0001	0.0001	0.00005
IL6	1.587 ± 0.061^a^	1.207 ± 0.023^b^	1.2 ± 0.026^b^	1.133 ± 0.054^b^	0.0072	0.0146	0.1505
IL10	1.39 ± 0.04^a^	0.600 ± 0.026^b^	0.367 ± 0.02^c^	0.317 ± 0.020^c^	0.0001	0.0001	0.00003
iNOS	1.747 ± 0.017^a^	0.933 ± 0.039^b^	0.583 ± 0.02^c^	0.450 ± 0.013^c^	0.0001	0.0001	0.00003
NF-kB	1.520 ± 0.032^a^	0.847 ± 0.014^b^	0.633 ± 0.015^c^	0.547 ± 0.014^c^	0.0001	0.0001	0.00002
TNF-α	1.273 ± 0.017^a^	0.847 ± 0.014^b^	0.633 ± 0.015^c^	0.547 ± 0.014^c^	0.0001	0.0001	0.0001

± : standard error means

^a–c^Means with different superscripts in the same row indicate significant differences (*P* < 0.05)

*IL* interleukin, *iNOS* inducible nitric oxide synthase, *NF-κB* nuclear factor kappa, *TNF-α* tumor necrosis factor

## Discussion

The most difficult period in the lives of lambs is undoubtedly the suckling period. During this period, lambs are especially vulnerable to unsuitable environments and pathogens, and their defense mechanisms are not yet sufficiently developed. Since lambs are exposed to an environment full of new and pathogenic microorganisms from the sterile uterine environment at birth, they have to adapt to this environment as soon as possible in order to survive [32, 33]. It has been proven by studies conducted in various animal species such as laboratory animals, cattle, broiler chickens, laying hens, pigs, and fish that boron supplements have important functions in the organism. However, in the literature review, no study was found regarding the use of boron in suckling lambs. In this study, we aimed to examine the effect of boric acid, a cheap boron source, on various systems during the suckling period, which is important for lambs.

Previous studies on the use of boric acid in various animals have demonstrated a varying effect on the performance of animals. It was determined that the groups significantly differed for AWWG. When compared to the control group, especially group B60 had a better AWWG. Additionally, it was observed that FCR was better in the B60 and B90 groups compared to the other groups. In a previous study on the use of sodium borate in 5 to 6-month-old lambs fed on a low-calcium diet, it was reported that boron had no effect on slaughter weight, but affected body weight gain [34]. In another study on the addition of different levels of boric acid (0, 100, 200, and 400 mg/L) to the drinking water of broiler chickens, it was determined that body weight gain was negatively affected in groups that received > 100 mg/L of boric acid [35]. It has been reported that the effects of boron on the feed conversion ratio vary in goats [36], broilers [17], laying hens [37], quails [38], pigs [39], and fish [40, 41].

It was determined that while the serum triglyceride level of group B30 was lower than that of the control group, there was no difference between groups B60 and C. Serum triglyceride levels increased with increased dietary boric acid levels. In a previous study conducted in dairy cows in the periparturient period, it was reported that serum triglyceride levels decreased with dietary sodium borate supplementation [42]. In a study on the effects of dietary boric acid in broiler chickens, it was determined that the serum triglyceride levels of the control subjects and the animals that received boric acid supplementation did not differ [19].

The highest TP level was observed in group C. In particular, the total protein level of group B60 was found to be significantly lower than that of group C. This result could be interpreted as groups C and B90 having been affected more severely by stress after birth, and the addition of 30 mg/kg BW and 60 mg/kg BW of boric acid to the milk replacer appeared to reduce stress. In a previous study on the effects of dietary boric acid supplementation on biochemical parameters in broiler chickens, it was reported that the TP levels were lower in the groups supplemented with boron, compared to the group that did not receive supplementation [43]. On the other hand, in a study conducted in female rabbits, it was reported that TP levels were high in the groups supplemented with boric acid, and this was associated with an increase in the globulin level [44].

ALP is known to be found at higher levels in young ruminants, compared to adults, due to osteoblastic activity [45]. In the present study, in which the effect of boric acid was investigated of lambs in suckling period, it was determined that serum ALP levels were higher in group B30 than in group C. It has been reported that boron has positive effects on Ca and P metabolism and improves bone health [46–48]. In a previous study on the effects of dietary sodium tetraborate on serum biochemical parameters in buffaloes, it was determined that serum ALP levels increased with dietary boron supplementation [49]. As indicated above, previous research on the effects of boron on serum ALP levels suggest that this effect may be related to osteoblast health and Ca-P metabolism.

Adaptation to the environment and the establishment of physiological balance both induce oxidative stress. Thus, the immune system weakens in lambs during the suckling period. During this period, it is beneficial to provide lambs with supplements to support their antioxidant system [50]. In the present study, it was determined that boric acid (B30, B60, and B90), a boron derivative with antioxidant properties, significantly increased TAS levels of lambs in the suckling period, compared to group C. TOS levels were found to be lower in the groups that received boric acid, compared to group C. The group with the highest TAS level and the lowest TOS level was group B60. In a study investigating the protective effect of boric acid on ethanol-induced kidney damage in rats, it was reported that TOS levels decreased and TAS levels increased with boric acid administration [51]. In another study on the effectiveness of boric acid against ischemic kidney damage in rats, it was reported that while TAS levels increased with increasing doses of boric acid (200 mg/kg), TOS levels were lower in rats given 50 and 100 mg/kg of boric acid [14]. Based on literature reports and the results of the present study, we suggest that dietary supplementation with boric acid may have positive effects on the antioxidant system of lambs in suckling period.

In the present study, serum MDA levels were compared and the highest were determined in group C. It is vital that the oxidant-antioxidant balance is in favor of antioxidants in pre-weaned lambs, as they are faced with many stress factors during the suckling period and damage that occurs during this period can negatively affect. In previous research, boron has been reported to show positive effects on the antioxidant system [52, 53]. In a study on the antioxidant levels of rats supplemented with boric acid and sodium tetraborate as different boron sources in their diets, it was reported that boron derivatives improved serum and tissue MDA levels, compared to the control subjects [52]. In another study on the effect of boric acid on the antioxidant system in rats, it was determined that serum MDA levels were lower in the group supplemented with boric acid against cisplatin toxicity, compared to the group not supplemented against cisplatin toxicity [53]. Based on literature information and the results of the present study, we hypothesize that MDA-induced cellular damage and lipid peroxidation can be reduced of lambs in the suckling period with dietary boric acid supplementation.

Literature review demonstrated that there is no previous study on the effect of boric acid supplementation on serum GSH levels of lambs in the suckling period. In the present study, in which boric acid was added to the milk replacer feed of lambs during the suckling period, it was shown that GSH levels had significantly increased (*P* < 0.05) in groups B30, B60, and B90, compared to group C. To date, studies on the effect of boric acid and other boron derivatives on GSH levels have been conducted mostly in laboratory animals [14, 54, 55]. In some of these studies, it was reported that GSH levels increased in response to the effect of boric acid [14, 55]. However, in a study conducted in rats, lower GSH levels were detected in groups with necrotic enterocolitis compared to the controls [54]. Based on literature reports and the results of the present study, we suggest that the addition of boric acid to the milk replacer feed of lambs during the suckling period may increase serum GSH levels and may provide benefits against stress associated adverse events effects caused by intense stress during this period.

The intestinal microbiota, the largest and most complex mammalian ecosystem, serves as a bridge between nutrients and animals and regulates body health [56]. It is closely related to the normal physiology of the animal, such as nutritional status, behavior, and stress [57]. Bacterial enteritis in lambs is a serious disease that affects weight gain and causes economic losses [58]. Diarrhea is one of the most commonly reported diseases in newborn ruminants. Studies have shown that the main pathogenic bacterial species responsible for diarrhea is *Escherichia coli* (*E.coli*), which belongs to the group of coliform bacteria [59]. Another bacterial pathogen that impairs intestinal health in lambs and sheep is *C. perfringens*. Clostridia are among the first bacterial communities to settle in the intestines of newborn animals [60]. Most of the enteric *C. perfringens* infections are referred to as enterotoxemia and cause serious losses in lambs and sheep. They multiply rapidly in the intestines under adverse conditions and produce toxins [61]. In the present study, the effect of boric acid on the fecal culture was one of the parameters investigated. Total coliform bacteria, *Clostridium*, and total bacteria counts were made in the collected fecal samples. According to the results obtained, there were significant differences between group C and the groups that received dietary boric acid for total coliform bacteria counts in parallel with the increase in the dose of dietary boric acid given. While the total coliform bacteria count was highest in group C, the lowest total coliform bacteria count was detected in group B90. This indicates that boric acid may have an inhibitory effect on the proliferation of total coliform bacteria. Differences were also observed between the study groups for the *Clostridium* counts in the fecal samples of the lambs. While the highest *Clostridium* count was determined in group C, the lowest *Clostridium* count was determined in groups B60 and B90. These results show that boron may be a candidate for use as a feed additive in protection studies against enterotoxemia. To the authors’ knowledge, there is no previous study on the effectiveness of boric acid supplementation on the fecal culture in lambs, and available studies have been conducted mostly in pet and laboratory animals and humans. In a study investigating the effectiveness of boric acid in the prevention of skin infections in dogs, it was reported that there was no significant difference between the group cleaned with boric acid-containing cleaning cloths and the control group [62]. In a laboratory study aimed at determining the antimicrobial activity of boron against *E. coli*, it was reported that *E. coli* counts decreased with the use of boric acid [16]. Literature review showed that there is no previous study on the effect of boron derivatives, especially boric acid, against clostridia. In the present study, which investigated the effect of boric acid on the fecal culture of lambs in the suckling period, it was determined that the coliform bacteria (*E. coli*) and *Clostridium* (*C. perfringens*) counts were reduced by boric acid. However, the total number of bacteria was increased by boric acid treatment. It was determined that the total number of bacteria had increased, and the highest numbers of bacteria were determined in groups B30, B60, and B90.

Closely related cytokines, such as TNF-α, NF-κB, IL-1β, IL-6, IL-10, and iNOS, play a role in the immune response. The stimulation of the immune system activates these cytokines [63]. These cytokines in the organism are always in relationship with each other. These cytokines, which increase in cases of stress and inflammation in the body, can prevent the damage of reactive oxygen species. The suckling period is one of the stressful periods in the lives of lambs. These stress conditions can trigger TNF-α production in the body and cause activation of NF-κB and iNOS [64, 65]. In response, the body increases the expression of genes such as IL-1β and IL-6. These cytokines were expressed less in the groups supplemented with boric acid compared to those in group C, and it can be said that boron has a positive effect against stress during the suckling period. According to the data obtained, the expression of these genes was statistically increased in group C, compared to the boric acid-treated groups. The differences observed between group C and boric acid treatments for the expression of IL-1β, IL-6, IL-10, iNOS, NF-kB, and TNF-α were statistically significant. As is the case with many of the other parameters investigated in the present study, to the authors’ knowledge, there is no previous study on the effect of boric acid and other boron derivatives on the expression of genes of lambs in the suckling period. Available studies have been conducted mostly in laboratory animals. In a study on lymphocyte proliferation in the mouse spleen, it was reported that boric acid increased the expression of TNF-α, IL-1β, IL-6, and iNOS [66]. In another study conducted in rats with calcium deficiency, it was stated that the expression of TNF-α increased due to the effect of boron [21]. It has also been reported that sodium borate supplementation increased TNF-α expression in cattle vaccinated against bovine herpes virus type 1 and that expression did not change by the 4th day after vaccination [67]. On the other hand, in a study on photoaging in rats, COX-2, IL-8, NF-kB, IL-6, and TNF-α expressions were determined to have decreased by the effect of sodium perborate tetrahydrate [68]. Given that the gene expressions of the boric acid-treated groups were lower than those of group C suggests that boron reduces inflammation under conditions of stress.

The results of the current study showed that the use of boric acid improved AWWG and FCR in lambs. In addition, in this study, it was observed that the use of boric acid had positive effects on the antioxidant system, fecal flora, and immune system-related gene expressions. In enterprises where milk replacer/milk is given to animals via feeding bottles or multiple feeding systems, boric acid can be easily added to the milk replacer and offered for consumption by the animals. More studies are needed to determine the effects of boric acid and other boron derivatives on the gastrointestinal system of lambs in the suckling period.

## Data Availability

The data that assist the results of the study are available from the corresponding author upon reasonable request.
